# Food supply, nutrition and trade policy: reversal of an import ban on turkey tails 

**DOI:** 10.2471/BLT.17.192468

**Published:** 2017-08-23

**Authors:** Anne Marie Thow, Erica Reeve, Take Naseri, Tim Martyn, Caroline Bollars

**Affiliations:** a Menzies Centre for Health Policy, D17 Charles Perkins Centre, University of Sydney, New South Wales, 2006, Australia.; b Centre for Population Health Research, Deakin University, Melbourne, Australia.; c Ministry of Health, Apia, Samoa.; d Centre for Pacific Island Studies, University of the Sunshine Coast, Queensland, Australia.; e Faculty of Health, Medicine and Life Sciences, Maastricht University, Maastricht, Netherlands.

Turkey tails are a cheap, fatty meat, imported into many developing countries. The Government of Samoa implemented an import ban on turkey tails in 2007 as part of its efforts to reduce noncommunicable diseases in the country.^1^ The population of Samoa has some of the highest rates of noncommunicable diseases in the world: in 2013, diabetes prevalence was 20% among adults and obesity prevalence was 53% among men and 77% among women.^2^ Dietary change associated with the nutrition transition – characterized by a shift from traditional diets high in cereal and fibre to diets higher in processed foods, sugars, fat and animal-source food – has been a significant contributor to the increase in prevalence of such diseases. In Samoa, as in many other low- and middle-income countries, trade liberalization and policies to promote exports have contributed to reducing the availability of traditional staples and increasing the availability of foods associated with the nutrition transition.^3^ The ban on turkey tails contributed to shifting consumption away from fatty meats: in a study conducted by the Samoan ministry of health in 2008, just under half of respondents reported that they consumed other cheap meats such as chicken, sausage or mutton instead of turkey tails, while about a quarter said they now ate lower-fat meat or seafood; only a few reported eating less meat overall as the result of the ban.^1^ Import data did not suggest a clear substitution of turkey tails with other fatty meats, including chicken, lamb or beef cuts.^1^

The Government of Samoa removed its ban on turkey tails as part of its accession to the World Trade Organization (WTO) in 2011. The removal of the ban illustrated the potential for international trade agreements to stifle innovation in nutrition policy-making through constraining policy space.^4^ Concerns have been raised globally regarding the impact of trade agreements on health and nutrition through both the direct impact of trade in unhealthy foods and the constraints on adopting best-practice nutrition policy interventions.^5^ In this paper, we examine the rationale for the removal of the turkey tails ban as part of Samoa’s accession to the WTO as well as Samoa’s response to this removal. We also consider the opportunity for policy learning – regarding the design of food policy for the prevention of noncommunicable diseases – for other low- and middle-income countries facing a rising burden of diet-related such diseases.

The working party overseeing Samoa’s accession to the WTO raised two main concerns about the ban related to issues at the heart of WTO and other trade agreements.^6^ Although these concerns reflect key elements of WTO law, the fact that the issues were raised during an accession process rather than a formal dispute means that the removal of the ban does not constitute a formal application of WTO law under any specific agreement(s). Nevertheless, the concerns raised are helpful in understanding important considerations in the design of public health nutrition regulation in relation to trade law, and in particular, to the general agreement on tariffs and trade and the agreement on technical barriers to trade. 

The first concern was the effectiveness of the ban in improving diets and reducing noncommunicable diseases, as other WTO members “questioned the prohibition of a single food item in order to address the […] complex problem of obesity”^6^. This concern refers to the imperative to use the least trade-restrictive measure needed to achieve a policy objective, also called the necessity test. The second concern referred to the principle of non-discrimination: “The import ban […] was […] discriminatory, as there are many high-fat foods, imported and domestic, still available for purchase in Samoa.”^6^ A key issue here is the availability of like products, a trade term for similar commodities available in the market, which were not subject to the ban.

In response to these concerns, the Government of Samoa committed to removing the turkey tails ban. However, the Government also demonstrated the potential to reclaim policy space for nutrition policy. The final accession agreement enshrined both trade and health commitments into international trade law. It included a commitment to conduct a study on options to replace the ban on turkey tails and to implement a temporary 300% tax on them.^6^

The study on policy options to replace the turkey tails ban was conducted in 2015, supported by a partnership between the Samoan ministry of health, the World Health Organization and the Food and Agriculture Organization. The recommendations of the study were aligned to global and regional evidence-based and best-practice recommendations^7^ and addressed the concerns of the working party by emphasizing a non-discriminatory, transparent and comprehensive approach to improving diets and preventing noncommunicable diseases.^8^ The findings of the study were reviewed in consultation with the national working group on trade agreements, chaired by the deputy prime minister and including representatives from the ministries of trade, commerce, agriculture, finance and health, before recommendations were finalized. Recommendations included: (i) implementing non-discriminatory fiscal policy measures, in terms of both taxes and subsidies, to create incentives for healthy food production and consumption, based on a nutrient profiling model; (ii) increasing consideration of nutrition in investments for agricultural production; (iii) investing in improving the healthfulness of food sold in the informal sector, based on an initiative conducted in Singapore that included training and certifying vendors;^9^ and (iv) improving the healthfulness of public procurement and implementing a targeted fruit and vegetable support measure into social welfare benefits. 

The main strength of this approach in relation to WTO law, particularly in relation to the concerns raised by the members of the accession committee, is its comprehensiveness and scientific underpinning. The recommendations address the concerns about discrimination by considering all sources of fat and other unhealthy nutrients in the food supply in Samoa, and by suggesting a transparent and scientifically based categorization of foods – from healthier to less healthy – using a nutrient profiling system. In doing this, the recommendations are non-discriminatory with respect to trade, as they apply to both imported and domestically produced foods. Furthermore, the recommendations include strategies to improve the healthfulness of foods available in the informal sector, including fruit and vegetables and prepared foods. Foods obtained in the informal sector make a significant contribution to consumption, but in contrast to foods sold in the formal sector, particularly those that are processed and packaged, they tend to be more difficult to regulate.

Samoa has started implementing the study’s recommendations and in July 2016, the minister for finance in Samoa announced that: “In this budget we have […] introduced excises on sugar items and some salt products which are damaging the health of too many of our people.”^10^ Samoa’s experience of trade-related constraints on nutrition policy space and its ability to develop a strategic policy response highlights two important lessons that may be of interest to other members of the WTO and to countries party to other international trade agreements. The prevalence of diet-related noncommunicable diseases is rising globally, particularly in low- and middle-income countries, and governments are actively seeking to increase access to healthy and affordable food through nutrition policies. However, trade agreements are increasingly including commitments, usually aimed at harmonization, that potentially constrain policy-making behind the border, that is, domestic policy-making that affects traded goods. These common global challenges indicate the potential for policy lessons arising from this case study.

First, Samoa’s experience highlights that no country is immune from trade constraints on nutrition policy-making, no matter how small the market or low the income. However, pro-active consideration of non-discrimination and necessity in the design of nutrition policy measures can help to minimize trade concerns. The study highlighted the benefits of (i) transparency in the scientific method used to identify unhealthy and healthy foods, for example, using a nutrient profiling tool; (ii) strategically designing a nutrition policy package rather than implementing one-off policy interventions; and (iii) including policies that address the nutritional quality of foods in both the formal and informal sectors, as the informal food sector is significant in most developing countries.^11^ This could include the use of standards to define healthy and unhealthy foods.

Second, public health actors must engage strategically with international trade and bilateral negotiations to improve health outcomes. Samoa’s decision to include a nutrition policy response as part of the WTO accession agreement meant that Samoa would be required to report to the WTO on the study, and it thus ensured buy-in from the economic sectors. The engagement of the ministry of health in the accession working party and national trade committee helped to get nutrition on the trade agenda,^12^ and the commitment of development partners was critical in harnessing the necessary cross-disciplinary expertise for the study.

The relevance of Samoa’s experience for other countries also needs to be considered in light of the fact that this trade and health outcome was negotiated during Samoa’s accession to the WTO. This process is different from the negotiation of other free trade agreements. Components of Samoa’s experience, such as the inclusion of commitments on compensatory health measures, may be relevant mainly to the accession context, because it is not tied to a specific WTO agreement. However, other factors are likely to be relevant to trade agreements more broadly. These factors include the benefits of health-sector participation in negotiations; the importance of a robust, scientific basis for policy intervention; consideration of the principles of non-discrimination and necessity in the design of nutrition policy; and the need to raise awareness among economic policy-makers on the importance of protecting policy space for nutrition.

Trade agreements are increasingly focused on harmonizing national measures affecting traded goods – and food is one of the most highly traded goods globally. Continued research is needed to support public health policy-makers to both identify and share strategies to mitigate the risks, inherent in binding global trade agreements, to policy space for nutrition. Policy-makers also need to enhance policy coherence between trade and health and to strengthen nutrition policy in an era of trade liberalization. Global nutrition agencies can also play an important role in (i) supporting the development of nutrition policy interventions with a strong scientific basis; (ii) working towards robust international standards to serve as the basis for nutrition policy development, in line with the commitments on Technical Barriers to Trade; and (iii) addressing policy coherence between trade and public health at the global level.
